# Using Magnetic Micelles Combined with Carbon Fiber Ionization Mass Spectrometry for the Screening of Trace Triazine Herbicides from Aqueous Samples

**DOI:** 10.3390/molecules29010137

**Published:** 2023-12-26

**Authors:** Chih-Wei Chen, Tzu-Ling Yang, Yu-Chie Chen

**Affiliations:** 1 Department of Applied Chemistry, National Yang Ming Chiao Tung University, Hsinchu 300, Taiwan; 2 International College of Semiconductor Technology, National Yang Ming Chiao Tung University, Hsinchu 300, Taiwan

**Keywords:** triazine herbicides, magnetic probes, magnetic metal ions, sodium dodecyl sulfate, carbon fiber ionization

## Abstract

Triazine herbicides are commonly used in agriculture to eliminate weeds. However, they can persist in the environment. In this study, we explored a new method for detecting triazine herbicides in aqueous samples. We selected two triazine herbicides, namely, prometryn and ametryn, as model herbicides. To generate magnetic probes, we mixed aqueous Gd^3+^ with aqueous sodium dodecyl sulfate (SDS), which created magnetic probes made of Gd^3+^-SDS micelles. These probes showed a trapping capacity for the model herbicides. Results indicated that the trapping capacities of our magnetic probes for ametryn and prometryn were approximately 466 and 468 nmol mg^−1^, respectively. The dissociation constants of our probes toward ametryn and prometryn were 2.92 × 10^−5^ and 1.27 × 10^−5^, respectively. This is the first report that the developed magnetic probes can be used to trap triazine herbicides. For detection, we used carbon fiber ionization mass spectrometry (CFI-MS), which can be used to directly detect semi-volatiles from the samples in the condensed phase. Because of the semi-volatility of triazine herbicides, the herbicides trapped by the magnetic probes can be directly analyzed by CFI-MS without any elution steps. In addition, we also demonstrated the feasibility of using our approach for detecting triazine herbicides in lake water and drinking water.

## 1. Introduction

Pesticides are commonly used in agriculture to prevent the growth of pests and weeds. Approximately three million tons of pesticides are consumed globally each year [1], but they pose concerns about health and environmental risks [2,3,4,5,6,7,8,9,10,11]. Ametryn, another triazine herbicide, has moderate toxicity for fish, mammals, and humans, but it has high toxicity to crustaceans and mollusks [9]. Poisoning by ametryn can cause vomiting, diarrhea, muscle weakness, and salivation, and long-term exposure can lead to cancer [10]. Ametryn can persist in soil for months and even years and can spread in soil vertically and horizontally due to its high-water solubility [11]. The maximum residue limit (MRL) for ametryn in drinking water regulated by Canada is 7 µg L^−1^ [12]. Prometryn, another triazine herbicide, is commonly used in growing crops such as cotton or celery to eliminate weeds [13]. According to the United States Environmental Protection Agency (US-EPA), the MRL for prometryn in groundwater is 23.2 µg L^−1^ (9.62 × 10^−8^ mole L^−1^) [14]. Therefore, developing analytical methods to quickly detect the presence of triazine herbicides in complex samples is crucial.

Gas chromatography [15,16,17,18], high-performance liquid chromatography [19,20], and surface-enhanced Raman spectroscopy [21] are commonly used to detect pesticides, including herbicides. However, pesticides are often found in complex sample matrices, which necessitates sample pretreatment prior to analysis using the abovementioned techniques [15,16,17,18,19,20,21,22]. The sample preparation steps and fabrication of required materials, such as adsorbents, should be straightforward and require minimal preparation time. While carbon nanotubes (CNTs) have been employed as effective adsorbents for trapping pesticides from environmental water, such as seawater and reservoir water, the fabrication of CNTs for this purpose is complex and time-consuming. Specifically, the CNTs need to be refluxed with acid for 8 h at 500 °C to oxidize the surface of the CNTs and generate negatively charged functional groups [23]. Additionally, biochar produced from corn straw has been utilized to remove triazine pesticides [24], but it requires heating at 500 °C for 2 h followed by a 12 h rinse with distilled water. Therefore, there is still a need to develop faster and simpler methods to fabricate suitable adsorbents for trapping target herbicides.

Magnetic adsorbents have gained significant attention due to their ease of isolation [25,26,27]. For instance, graphene oxide modified with dopamine and Fe_3_O_4_ magnetic nanoparticles has been investigated for trapping triazine herbicides [28]. The modification of graphene oxide requires heating at 60 °C for 12 h. Similarly, magnetic poly(divinylbenzene-co-N-vinylpyrrolidone) nanoparticles, composed of polymers and magnetic materials, have been used as effective adsorbents for extracting triazine herbicides from environmental water [29]. The generation of such functional magnetic probes usually takes several hours [28,29]. Therefore, simplifying the fabrication steps for functional magnetic probes is desirable. We have previously demonstrated that non-magnetic species, such as bacteria and cells, can gain magnetism by attaching abundant magnetic metal ions such as Fe^3+^, Co^2+^, Ni^2+^, and Gd^3+^ [30,31]. This is because increasing the density of unpaired electron spins in the conjugates greatly increases the magnetic susceptibility, resulting in manipulable and visible magnetism [30]. Surfactants are amphiphilic molecules that can form micelles at concentrations called critical micelle concentration (CMC). For example, the CMC of sodium dodecyl sulfate (SDS), an anionic surfactant, is approximately 8 mM [32]. Magnetic metal ions, such as gadolinium ions and ferric ions, can easily bind to the surface of SDS-formed micelles through electrostatic interactions [33]. As a result, the unpaired electron spins from the surface of the surfactant micelle–magnetic metal ions should be greatly enhanced, leading to the conjugates gaining magnetism to a certain extent. Triazine herbicides carry net positive charges at a low pH [34]. Therefore, positively charged triazine herbicides should be able to interact with anionic sites on the SDS micelle by removing Gd^3+^ from the conjugates. This makes magnetic metal ion-SDS micelles suitable as trapping probes for triazine herbicides.

Carbon fiber ionization mass spectrometry (CFI-MS) [35,36,37] is a simple and effective tool for detecting volatiles or semi-volatiles derived from samples in either solid or liquid form at atmospheric pressure. The ionization method in CFI-MS requires only a thin and short carbon fiber (diameter: ~10 μm; length: ~1 cm) placed near the inlet of the mass spectrometer [35]. Samples can be placed directly under the carbon fiber, and the volatiles or semi-volatiles derived from the samples can be directly ionized and detected simultaneously for CFI-MS analysis [37]. Another advantage of CFI-MS is its low background interference. Since polar species cannot be evaporated, species with high polarities do not interfere with the analysis of volatiles or semi-volatiles when using CFI-MS for the direct detection of analytes in the gas phase. Gd^3+^-SDS conjugates are non-volatile. Therefore, after the non-volatile probes trap target semi-volatile triazine herbicides, the probe–triazine herbicide conjugates can be directly placed underneath the carbon fiber in the CFI-MS setup without the need to elute target analytes to eliminate background interferences. In this study, the feasibility of coupling the use of Gd^3+^-SDS conjugates as the probes and using CFI-MS as the detection tool for model triazine herbicides was demonstrated.

## 2. Experimental Section

### 2.1. Chemicals and Materials

Gadolinium (III) chloride hexahydrate was purchased from Alfa Aesar (Ward Hill, MA, USA). Sodium dodecyl sulfate was purchased from Mallinckrodt (St. Louis, MO, USA). Ametryn, bovine serum albumin (BSA), cobalt (II) chloride hexahydrate, ferric chloride hexahydrate, L-cysteine, prometryn, and sodium acetate were purchased from Sigma-Aldrich (St. Louis, MO, USA). Manganous chloride, hydrochloric acid (36.5–38%), tris(hydroxymethyl)aminomethane, and tris hydrochloride were purchased from J. T. Baker (Phillipsburg, NJ, USA). Copper(II) chloride-2-hydrate was purchased from Riedel-de Haen (Seelze, Germany). Acetonitrile was obtained from Merck (Darmstadt, Germany). Methanol and ethanol (>99.5%) were obtained from Echo (Miaoli, Taiwan) and Macron (Center Valley, PA, USA), respectively. Deionized water was obtained from Taisun (Changhua, Taiwan). Magnets (~4000 Gauss) were obtained from a local company. Carbon fibers derived from carbon fabrics were purchased from a local shop.

### 2.2. Instrumentation

All mass spectra were obtained by using a micrOTOF Q II mass spectrometer (Bruker Daltonic, Bremen, Germany) at the positive ion modes. The voltage of −4500 V was applied, respectively, on the orifice of the mass spectrometer. The ion transfer capillary temperature was set to 200 °C, and the nebulizer was turned off. UV-Vis absorption spectra were collected using a Varian Cary 50 UV-Vis absorption spectrophotometer (Palo Alto, CA, USA), and fluorescence spectra were obtained using a Horiba Jobin Yvon Fluoromax-3 spectrofluorometer (Edison, NJ, USA). A superconducting quantum interference device (SQUID) (MPMS XL-7) (San Diego, CA, USA) was used to obtain the magnetism hysteresis curves of our magnetic adsorbents.

### 2.3. Synthesis of the Magnetic Gd^3+^-SDS Conjugates

The magnetic conjugates comprising Gd^3+^ and SDS were prepared by mixing 0.1 mL of aqueous GdCl_3_ (0.1 M) and 0.1 mL of SDS (20 mM) at room temperature, under shaking at 500 rpm for 10 min based on a previous study [33]. The magnetic conjugates were isolated by applying an external magnet with a strength of ~4000 Gauss. The collected magnetic conjugates were used as the adsorbents in this study.

### 2.4. Optimization of Experimental Parameters

The optimal incubation temperature, pH, the amounts of adsorbents, and the incubation time were determined. To examine the optimal temperature, Gd^3+^-SDS conjugates (~0.2 mg) were used as adsorbents to trap prometryn (1 mL, 0.2 mM) in sodium acetate buffer (20 mM) at a pH of 4, under shaking at 1000 rpm for 1 h, at temperatures ranging from 5 °C to 45 °C. The optimal pH was determined using similar parameters, with the buffers at pH levels of 3 to 9, and the incubation temperature set at 5 °C. Samples prepared at pH levels of 3, 4, 5, and 6 were placed in a sodium acetate buffer, while samples at pH levels of 7, 8, and 9 were placed in a Tris buffer. The extraction times varied from 10 to 60 min, with the pH and incubation temperature set at a pH of 4 and 5 °C, respectively. The optimal parameters were used for subsequent analysis in this study.

### 2.5. Examination of Adsorption Isotherms of the Gd^3+^-SDS Adsorbents against Triazine Herbicides

The binding affinity of Gd^3+^-SDS adsorbents against two model triazine herbicides was investigated by batch experiments. Initially, prometryn and ametryn at different concentrations were prepared in sodium acetate buffer (20 mM, pH 4). The Gd^3+^-SDS adsorbents (~0.2 mg) were added to the sample (1 mL) at a pH of 4 and incubated under shaking at 1000 rpm for 1 h at 5 °C, followed by magnetic isolation. The resultant supernatant was analyzed by UV-Vis absorption spectroscopy to record the absorbance changes of the maximum absorption bands of the samples obtained before and after being trapped by the adsorbents, and the trapping capacity was estimated accordingly. The dissociation constant (*k_d_*) was determined by fitting the obtained curve using the Hill equation [38].

### 2.6. Using CFI-MS as the Detection Tool

Prometryn and ametryn were used as model herbicides. Samples containing the herbicides at given concentrations (10^−8^ M to 10^−9^ M) were prepared in an acetic buffer (20 mM) at a pH of 4. Gd^3+^-SDS adsorbents (~0.2 mg) were added to each sample (1 mL), which were then shaken at 1000 rpm at 5 °C for 2 h. The resulting conjugates were isolated by placing an external magnet next to the sample vial to remove the supernatant, followed by resuspension in acetonitrile (20 µL). The resulting sample was directly analyzed by CFI-MS [37]. A metal inlet (inner diameter ~1 mm, outer diameter ~1.5 mm) with a length of 4 cm was attached to the orifice of the mass spectrometer. A piece of thin carbon fiber (length: ~1 cm; diameter: ~10 μm) held by a pair of wooden tweezers was placed approximately 6 mm away from the inlet of the mass spectrometer and applied with a voltage of −4500 V. After switching on the mass spectrometer, the sample was placed under the carbon fiber with 0.5 cm. Mass spectra were acquired immediately after the sample was placed under the carbon fiber.

### 2.7. Examination of Interference Species

To evaluate the effect from the interference species, interference species, including metal ions and biomolecules, were added to the samples. Specifically, a sample containing a model herbicide (1 or 10 nM) prepared in acetic buffer (20 mM) at a pH of 4 was added with metal ions (10 μM) such as Fe^3+^, Cu^2+^, Mn^2+^, and Co^2+^. Another sample containing a model herbicide (1 or 10 nM) prepared in an acetic buffer (20 mM) at a pH of 4 was added with L-cysteine (1 μM) and BSA (1 μM). The sample pretreatment step using Gd^3+^-SDS conjugates as the adsorbents against target analytes followed by CFI-MS analysis was carried out as described above.

### 2.8. Analysis of Simulated Real Samples

Lake water collected from our campus and drinking water from a drinking water dispenser in our building were used as real samples. The collected water sample (0.5 mL) was spiked with prometryn (10^−7^ M) and mixed with an acetic buffer (0.5 mL, 20 mM, pH 4), followed by shaking with Gd^3+^-SDS adsorbents (~0.2 mg) at 1000 rpm for 1 h at 5 °C. The resultant adsorbent–target species conjugates were collected via magnetic isolation. Acetonitrile (20 µL) was added to the collected conjugates prior to CFI-MS analysis. The operational steps for MS analysis were similar to those stated above.

## 3. Results and Discussion

### 3.1. Using the Gd^3+^-SDS Conjugates as the Adsorbents for Trapping Herbicides

**Figure 1:**
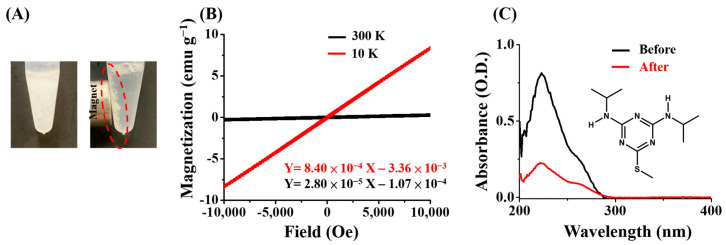
Examination of the magnetism of the Gd^3+^-SDS conjugates and their capacity to trap prometryn: (**A**) photographs of the Gd^3+^-SDS conjugates before (**left**) and after (**right**) placing an external magnet (~4000 Gauss) next to the vials. The red circle indicated where the magnetic conjugates are; (**B**) hysteresis curves of the Gd^3+^-SDS conjugates obtained at 10 (red) and 300 K (black); and (**C**) UV-Vis absorption spectra of the supernatants of the samples containing prometryn (400 μM) prepared in acetic buffer (20 mM) at a pH of 4 obtained before (black) and after (red) being incubated with the Gd^3+^-SDS conjugates (~0.2 mg). The inset shows the chemical structure of prometryn.

The Gd^3+^-SDS conjugates were used as adsorbents to trap triazine herbicides. Initially, the magnetism of the adsorbents was examined. Figure 1A shows photographs of vials containing the Gd^3+^-SDS suspension without (left) and with (right) an external magnet placed. It was observed that the Gd^3+^-SDS conjugates had visible magnetism and were attracted to the wall of the vial next to the external magnet (photograph on the right-hand side in Figure 1A). This indicated that the magnetic Gd^3+^-SDS conjugates were successfully generated. Figure 1B shows the corresponding SQUID magnetization loops with the magnetic field applied along the magnetization axis measured at 10 (red) and 300 K (black). The magnetic susceptibilities of the Gd^3+^-SDS precipitates were 8.40 × 10^−4^ emu g^−1^ and 2.80 × 10^−5^ emu g^−1^ at 10 and 300 K, respectively (Figure 1B). The magnetic conjugates were further used as adsorbents against a model herbicide, prometryn. The inset in Figure 1C shows the structure of prometryn. Figure 1C displays the absorption spectra of the supernatants of the samples containing prometryn (400 μM) obtained before (black) and after (red) incubation with the Gd^3+^-SDS adsorbents at a pH of 4, followed by magnetic isolation. It was observed that the maximum absorption band at 223 nm, derived from prometryn in the supernatant obtained after magnetic isolation (red band), was greatly decreased. This indicates that the Gd^3+^-SDS adsorbents were capable of trapping prometryn.

### 3.2. Optimization of Experimental Parameters

**Figure 2:**
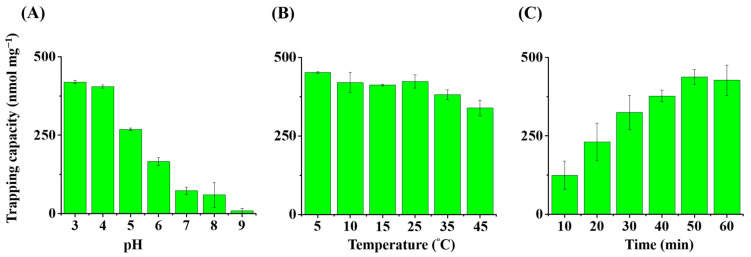
Examination of the optimal parameters: (**A**) bar graphs of the trapping capacity of the Gd^3+^-SDS adsorbents toward prometryn at different pH conditions. The samples (1 mL) containing prometryn (400 μM) prepared in acetic buffers at pH levels of 3, 4, 5, and 6 and Tris buffer (20 mM) at pH levels of 7, 8, and 9 were incubated with the Gd^3+^-SDS adsorbents (~0.2 mg) at 5 °C under shaking (1000 rpm) for 1 h; (**B**) bar graphs of the trapping capacity of the Gd^3+^-SDS adsorbents (~0.2 mg) toward prometryn (400 μM) in the sample (1 mL, pH 4) at different temperatures at 5 °C under shaking (1000 rpm) for 1 h; (**C**) bar graphs of the trapping capacity of the Gd^3+^-SDS adsorbents (0.2 mg) toward prometryn (400 μM) in the sample (1 mL, pH 4) incubated at 5 °C under shaking (1000 rpm) for different times under shaking at 10,000 rpm.

We conducted further examinations to determine the optimal pH value, the temperature, and the incubation time for trapping triazine herbicides using prometryn as the model analyte and the Gd^3+^-SDS conjugates as the adsorbents. Figure 2A presents a summarized histogram of the trapping capacity of prometryn on the adsorbents when the trapping experiments were carried out at different pH conditions (pH 3 to 9). The results showed that the adsorbents’ trapping capacity towards prometryn was ~400 nmol mg^−1^ at pH 3 and pH 4 and gradually decreased with an increase in pH in the sample solutions. As the pH increased above pH 7, the binding amount of prometryn to the adsorbents decreased. Prometryn carries a net positive charge in acidic conditions and can bind to the negatively charged SDS in the Gd^3+^-SDS conjugates by replacing Gd^3+^. Thus, we further examined whether the trapping mechanism was similar to what we proposed. Supporting Information (SI) Figure S1 shows the fluorescence spectra (λ_ex_ = 273 nm) of the supernatants of the samples containing the Gd^3+^-SDS conjugates mixed without (black) and with prometryn (red) obtained under shaking for 1 h followed by magnetic isolation. The emission band at the wavelength of 312 nm appearing in Figure S1 indicated the presence of Gd^3+^. Even when no analytes, such as prometryn, were present in the samples, Gd^3+^ from the Gd^3+^-SDS adsorbents could be released into the supernatant due to dynamic equilibrium. The emission band (red) at 312 nm derived from Gd^3+^ increased in the sample containing prometryn. The results indicated that prometryn replaced Gd^3+^ on the Gd^3+^-SDS conjugates, leading to a higher concentration of Gd^3+^ released in the supernatant. However, the remaining conjugates still possessed abundant Gd^3+^ and could be magnetically isolated by placing an external magnet.

We investigated whether the trapping results were affected by temperature in the trapping experiments. Figure 2B shows a summarized bar graph of the trapping capacity of the Gd^3+^-SDS conjugates towards prometryn obtained at different incubation temperatures. The highest trapping capacity was observed at 5 °C, although it did not vary much between temperatures of 10 °C and 35 °C. However, the trapping capacity decreased significantly when the temperature was increased to 45 °C. This decreasing in trapping capacity was presumably due to the dissociation of some Gd^3+^-SDS conjugates at higher temperatures, resulting in a weaker trapping capacity towards prometryn. To verify this hypothesis, two samples containing the same concentration of Gd^3+^-SDS conjugates were incubated at 5 °C and 45 °C under shaking at 1000 rpm for 1 h, followed by magnetic isolation. The resultant supernatants were examined by fluorescence spectroscopy. SI Figure S2 shows the fluorescence spectra of the supernatants of the sample containing the Gd^3+^-SDS conjugates obtained from the incubation temperatures of 5 °C (black) and 45 °C (red). The emission band derived from Gd^3+^ obtained from the sample incubated at 45 °C (red) had a higher intensity than that obtained at 5 °C (black), confirming our hypothesis that more Gd^3+^ was released to the supernatant at a higher temperature, presumably because more conjugates were dissociated at a higher temperature. Therefore, a lower binding capacity of prometryn on the Gd^3+^-SDS conjugates was obtained at a higher temperature, as shown in Figure 2B. In addition, we examined the optimal incubation time (Figure 2C), and the results showed that equilibrium was reached after incubating the sample with the adsorbent for approximately 1 h. Based on the results shown in Figure 2A–C, we determined that the optimal pH, incubation temperature, and time were pH 4, 5 °C, and 1 h, respectively. We used these optimal parameters for the following studies.

### 3.3. Examination of the Adsorption Isotherms of Triazine Herbicides on the Gd^3+^-SDS Adsorbents

**Figure 3:**
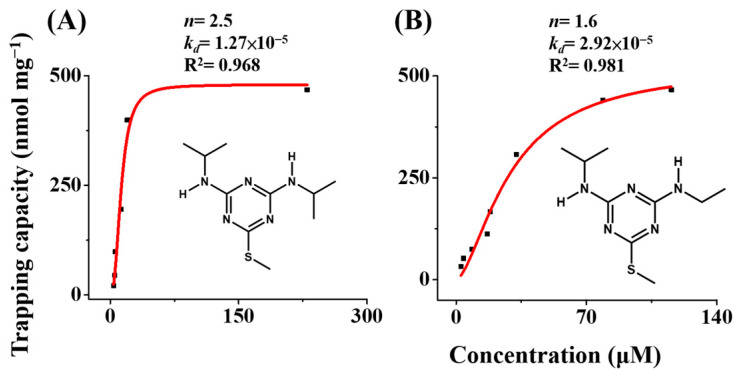
Examination of binding affinity. Adsorption isotherms obtained by plotting the trapping capacity of the Gd^3+^-SDS conjugates toward: (**A**) prometryn and (**B**) ametryn versus the concentration left in the supernatants of the samples.

To investigate further the binding affinity between the Gd^3+^-SDS adsorbents and triazine herbicides, their adsorption isotherms were examined. In addition to prometryn, ametryn was also selected as the model herbicide. Figure 3A,B shows the adsorption isotherms of the Gd^3+^-SDS adsorbent against prometryn and ametryn, respectively, at 5 °C. The Hill equation [38] was used to fit the adsorption isotherms, and the corresponding *k_d_* of prometryn and ametryn towards the adsorbents were 1.27 × 10^−5^ and 2.92 × 10^−5^, respectively. Moreover, the *n* values for prometryn (*n* = 2.5) and ametryn (*n* = 1.6) were larger than 1, indicating cooperative binding involved in the trapping processes. These facts were also reflected in the binding capacity of the Gd^3+^-SDS adsorbents towards these model herbicides. These results demonstrate that the Gd^3+^-SDS adsorbents can be used as suitable trapping probes to bind with the model herbicides.

### 3.4. Using CFI-MS as the Detection Tool

**Figure 4:**
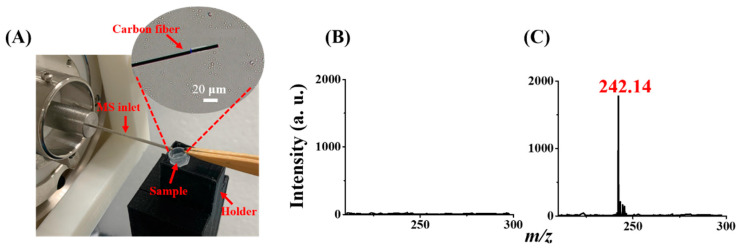
CFI-MS setup and the MS results: (**A**) photograph of the sample placed on a holder under the carbon fiber in the CFI setup. Direct CFI mass spectra of the samples (1 mL) containing prometryn with the concentration of 10^−7^ M prepared in acetic buffer (20 mM) at a pH of 4 obtained (**B**) before and (**C**) after being enriched by the magnetic Gd^3+^-SDS conjugates as the trapping probes followed by magnetic isolation. SRM mode was employed by monitoring the ion at *m*/*z* 242.14 (±1) with a collision energy of 0.1 eV.

We demonstrated that magnetic Gd^3+^-SDS conjugates could trap triazine herbicides, including prometryn and ametryn. Furthermore, we showed that it is feasible to use CFI-MS as a detection tool for direct analysis of the model herbicides trapped by the Gd^3+^-SDS adsorbents. CFI-MS has been proven to be a suitable detection tool for analyzing the vapor of volatile and semi-volatile analytes in their liquid or solid form [36]. Prometryn and ametryn are semi-volatiles, and their corresponding vapor pressures are 1.3 × 10^−6^ [39] and 2.7 × 10^−6^ [39], respectively. Therefore, CFI-MS can be used to easily detect the herbicides trapped by the magnetic adsorbents by simply placing the magnetic conjugates containing the target herbicides under the carbon fiber without conducting any tedious elution processes. Figure 4A shows the photograph of the CFI-MS setup, and Figure 4B shows the direct CFI mass spectrum of the sample (1 mL) containing prometryn (10^−7^ M) by placing the sample vial under the carbon fiber from the liquid surface to the carbon fiber. MS/MS mode was employed by monitoring the ion at *m*/*z* 242 with a collision energy of 0.1 eV. No ions derived from prometryn were observed. The sample was placed close to the carbon fiber. Figure 4C shows the resultant CFI mass spectrum of the same sample used in Figure 4B obtained after using the Gd^3+^-SDS conjugates as adsorbents followed by magnetic isolation and the addition of acetonitrile (20 μL) before CFI-MS analysis. The peak at *m*/*z* 242.14, derived from the protonated prometryn (monoisotopic mass of MH^+^ = 242.136 Da), appeared in the mass spectrum. These results indicate that our adsorbents can be used to trap prometryn, and the vapor derived from the adsorbent–prometryn conjugates can be readily detected by CFI-MS using our setup without further conducting elution steps.

**Figure 5:**
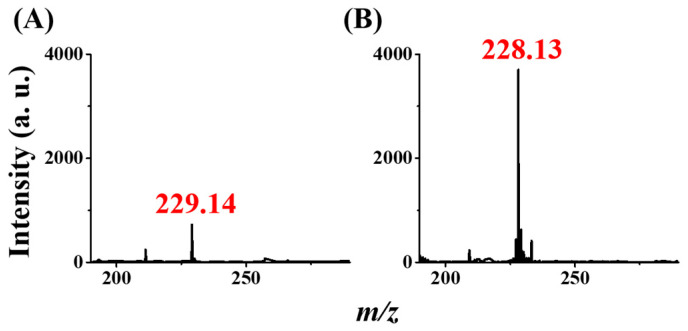
Using ametryn as the model sample. CFI mass spectra of the samples (1 mL) containing ametryn with the concentration of 10^−7^ M prepared in acetic buffer (20 mM) at pH 4 obtained (**A**) before and (**B**) after enriched by the magnetic Gd^3+^-SDS conjugates as the trapping probes followed by magnetic isolation. SRM mode was employed by monitoring the ion at *m*/*z* 228.13 (±1) with the collision energy of 0.1 eV.

Furthermore, we investigated whether our method could detect samples containing ametryn. Figure 5A,B shows the CFI mass spectra of the sample containing ametryn (10^−7^ M) obtained before and after enrichment, respectively, by the Gd^3+^-SDS adsorbents. A background peak at *m*/*z* 229 was observed before enrichment (Figure 5A). The mass spectrum shows a peak at *m*/*z* 228.13 derived from protonated ametryn (monoisotopic mass of MH^+^ = 228.12 Da) after enrichment (Figure 5B). These results demonstrate that our method can detect triazine herbicides.

### 3.5. Examination of the Effects of Interference Species

**Figure 6:**
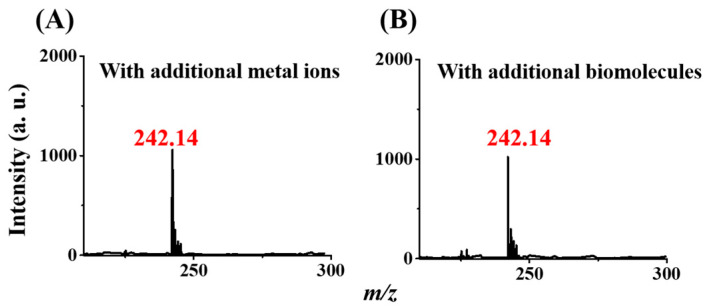
Effects from interference species. CFI mass spectra of the samples (1 mL) containing prometryn with the concentration of 10^−7^ M prepared in acetic buffer at pH 4 with the addition of (**A**) the mixture of the metal ions (5 μM) including Fe^3+^, Cu^2+^, Mn^2+^, and Co^2+^; and (**B**) the mixture of bovine serum albumin (5 μM) and cysteine (5 μM) by using the magnetic Gd^3+^-SDS adsorbents as the trapping probes followed by magnetic isolation and CFI-MS analysis.

To evaluate the effects of interference species, several common species that are typically present in real-world samples were examined. We added metal ions, such as Fe^3+^, Cu^2+^, Mn^2+^, and Co^2+^, as well as biomolecules, such as cysteine and BSA, to samples containing prometryn. Figure 6A,B displays the CFI mass spectra of the samples containing prometryn with the addition of the metal ion mixture and the biomolecules mixture, respectively, obtained using our method. The peak at *m*/*z* 242.14 derived from the protonated prometryn was still visible, although its intensity was slightly lower than that shown in Figure 4C. The presence of metal ions may have displaced Gd^3+^ in the Gd^3+^-SDS conjugates, resulting in a slight impact on our analysis. Moreover, cysteine and BSA have isoelectric points of a pH ~6 [40] and a pH ~4.7 [41], respectively. Thus, these two biomolecules carried a net positive charge at a pH of 4, the pH of the extraction condition in our method. These biomolecules with net positive charges may have competed with the negatively charged SDS with Gd^3+^ in the Gd^3+^-SDS adsorbents, causing an adverse effect on decreasing the ion intensity of the peak at *m*/*z* 242.14. Nevertheless, the sizes of the molecules are relatively large, leading to small effects on our results. Although these common interference species slightly affected the analysis of prometryn, the peak derived from the protonated prometryn was still observable in the resultant CFI mass spectra after enrichment by our method, as the concentration of prometryn was reduced to 10^−8^ M and 10^−9^ M according to our experimental results (SI Figure S3). The results indicated that the lowest detectable concentration was 1 nM, which was lower than the regulated MRL (i.e., 96.2 nM) by the US-EPA [14].

### 3.6. Analysis of Simulated Real Samples

**Figure 7:**
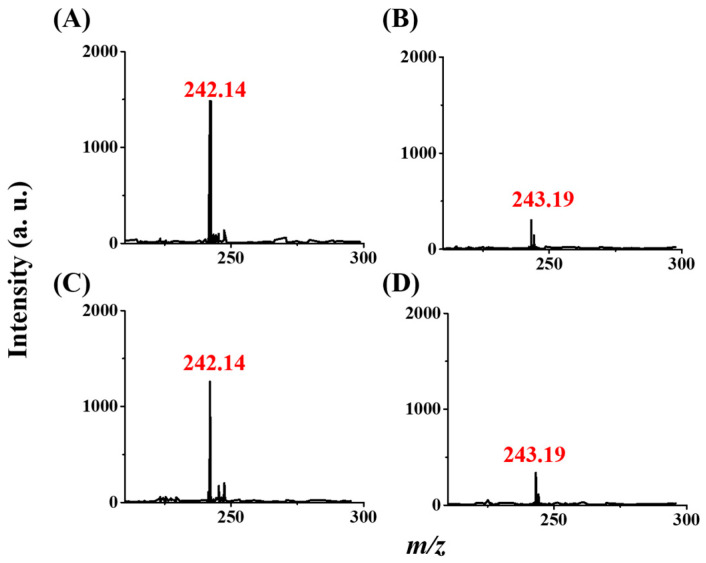
Analysis of the simulated real samples. CFI mass spectra of the drinking water samples (1 mL) spiked with prometryn with the concentration of (**A**) 10^−7^ and (**B**) 0 M. CFI mass spectra of the lake water sample (1 mL) spiked with prometryn (**C**) 10^−7^ M and (**D**) 0 M. Both samples were prepared in an acetic buffer at a pH of 4 obtained by using the magnetic Gd^3+^-SDS conjugates as the adsorbents followed by magnetic isolation and CFI-MS analysis.

Triazine can contaminate drinking water or lake water through accidents or the discharge of wastewater. Therefore, we selected drinking water and lake water as our actual samples. Figure 7A,B shows the resulting CFI-MS spectra of the drinking water spiked with prometryn (10^−7^ M) and without, respectively, obtained after using our method. Figure 7C,D shows the resulting CFI-MS spectra of the lake water spiked with prometryn (10^−7^ M) and without, respectively, obtained after using our method. The peak at *m*/*z* 242, derived from the protonated prometryn, is apparent in Figure 7A,C. The peak at *m*/*z* 243 was derived from background. The intensity at the peak derived from protonated prometryn was very similar to that obtained in Figure 4C, in which the sample was simply prepared in an acetic buffer. The results indicate that our method has the potential to screen the presence of such triazine herbicides in real-world samples.

## 4. Conclusions

Magnetic materials are widely used as affinity probes for trace analytes in complex samples due to their magnetic properties, making isolation easier. However, most existing synthesis methods for generating magnetic probes are complicated and require several hours in the synthesis steps. In this study, we successfully used magnetic probes that could be generated within 10 min by simply mixing Gd^3+^ and SDS adsorbents for trapping triazine herbicides. Due to the semi-volatility of triazine herbicides, CFI-MS was shown to be a suitable tool for direct analysis of the magnetic probe–triazine herbicides without the need for additional elution steps. Moreover, non-volatile interference species or the magnetic probes themselves did not cause any background in the resultant mass spectra due to their non-volatility. Although the trapping between the Gd^3+^-SDS conjugates and the model herbicides mainly resulted from electrostatic interaction, the selectivity and sensitivity of our approach were desirable when using CFI-MS as the detection tool. Owing to the short analysis time and low detectable concentration (i.e., ~1 nM), our approach can be used to rapidly screen the presence of trace triazine-herbicides in aqueous samples. While only triazine-herbicides were analyzed as target analytes in this study, we believe that the same probe combined with CFI-MS can be used to enrich various target species possessing volatility or semi-volatility through electrostatic interaction.

## Data Availability

Data are contained within the article and supplementary materials.

## Supplementary Materials

The following supporting information can be downloaded at: https://www.mdpi.com/article/10.3390/molecules29010137/s1, Figure S1: Fluorescence spectra of the supernatants of the samples containing the Gd^3+^-SDS adsorbent suspension mixed without and with the addition of prometryn; Figure S2: Examination of the temperature effects; Figure S3: Examination of the lowest detectable concentration.
