# Supplementary material for: Using Magnetic Micelles Combined with Carbon Fiber Ionization Mass Spectrometry for the Screening of Trace Triazine Herbicides from Aqueous Samples
Source: Molecules. 2023 Dec 26;29(1):137. doi: 10.3390/molecules29010137 (PMC10779876; doi:10.3390/molecules29010137)
Supplement: Supplementary file 1 [file molecules-29-00137-s001.zip › molecules-2773416-supplementary.pdf]

Supporting Information

Using Magnetic Micelles Combined with Carbon Fiber Ionization Mass Spectrometry for Screening of Trace Triazine Herbicides from Aqueous Samples

Chih-Wei Chen,[1] Tzu-Ling Yang,[1] and Yu-Chie Chen[*1,2]

[1]Department of Applied Chemistry, National Yang Ming Chiao Tung University, Hsinchu 300, Taiwan
[2]International College of Semiconductor Technology, National Yang Ming Chiao Tung University, Hsinchu 300, Taiwan

[*]Corresponding author
E-mail: yuchie@nycu.edu.tw
Tel: +886-3-5131527
Fax: +886-3-5723764

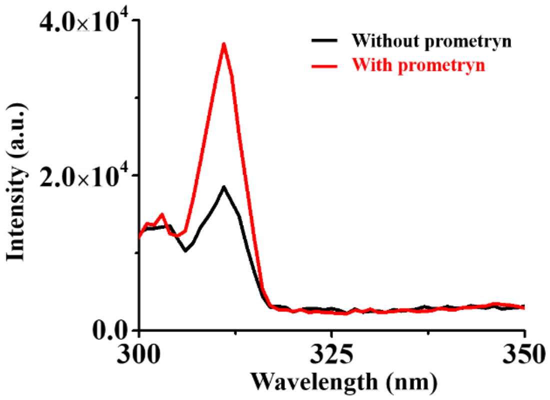

Figure S1. Fluorescence spectra (λ_{ex}= 273 nm) of the supernatants of the samples (1 mL) containing the Gd^{3+}-SDS adsorbent suspension (~0.2 mg) mixed without (black) and with (red) the addition of prometryn (400 µM) prepared in acetic buffer at pH 4 obtained after shaking at 1000 rpm for 1 h followed by magnetic isolation.

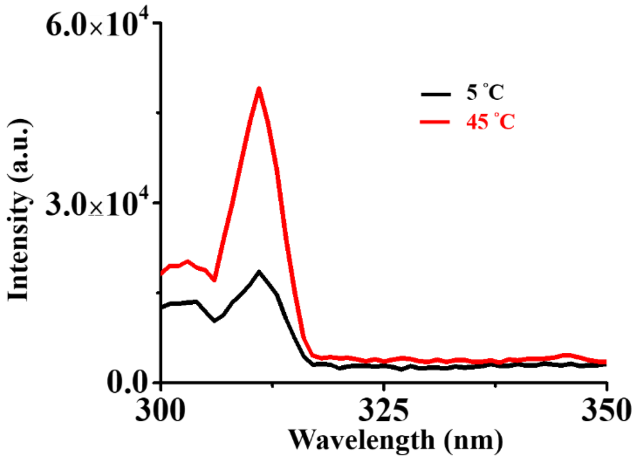

Figure S2. Examination of the temperature effects. Fluorescence spectra (λ_{ex}= 273 nm) of the supernatants of the samples (1 mL) containing the Gd^{3+}-SDS adsorbents (~0.2 mg) obtained at 5 °C (black) and 45 °C (red) under shaking at 1000 rpm for 1 h followed by magnetic isolation.

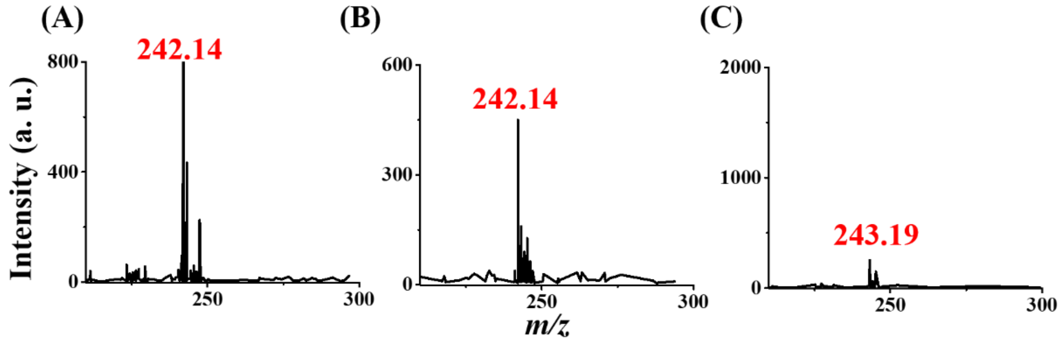

Figure S3. Examination of the lowest detectable concentration. CFI mass spectra of the samples containing prometryn with the concentrations of (A) 10^{-8}, (B) 10^{-9}, and (C) 0 M prepared in acetic buffer (1 mL) at pH 4 obtained after using the Gd^{3+}-SDS conjugates as the trapping probes followed by magnetic isolation. SRM mode was used by selecting the ion at *m/z* 242 as the target ion. Collision energy: 0.1 eV. The ion at *m/z* 243.19 in Panel C was the background ion.
